# Simultaneous Determination of Ten Bioactive Components from Shenling Baizhu San in Rat Plasma by UHPLC-MS/MS: Application to a Comparative Pharmacokinetic Study in Normal and Two Models of Ulcerative Colitis Rats

**DOI:** 10.1155/2021/3518241

**Published:** 2021-12-29

**Authors:** Xia Xu, Weiwei Wang, Yaxi Chen, Qiyun Zhang, Bingtao Li, Yiwen Zhong, Yu Tu, Wentong Zhang, Guoliang Xu, Li Jiang

**Affiliations:** ^1^Research Center for Differentiation and Development of CM Basic Theory, Jiangxi University of Chinese Medicine, Nanchang 330004, China; ^2^Department of Pharmacy, Affiliated Hospital of Jinggangshan University, Ji'an, Jiangxi 343000, China; ^3^Jiangxi Province Key Laboratory of CM Etiopathogenesis, Jiangxi University of Chinese Medicine, Nanchang 330004, China; ^4^Key Laboratory of Pharmacology of Traditional Chinese Medicine in Jiangxi, Nanchang 330004, China

## Abstract

Shenling Baizhu San, a traditional formula, has a long history of treating spleen asthenic diarrhea by invigorating the spleen and dispelling dampness in China. A rapid and accurate UHPLC-MS/MS method was developed and fully validated for the simultaneous determination of ten active constituents in rat plasma: panaxadiol, ginsenoside Rg1, atractylenolide I, atractylenolide III, pachymic acid, neferine, nuciferine, diosgenin, platycodin D, and isoliquiritigenin. The plasma samples were pretreated by the protein precipitation method with acetonitrile. The analytes and puerarin (internal standard) were determined with high selectivity and sensitivity (LLOQ, 0.31–0.68 ng·mL^−1^) within 10 minutes. The validation parameters, including intra-/interday precisions, accuracy, recovery, matrix effect, and stability, were within acceptable ranges. The validated method was successfully applied to the pharmacokinetics study of ten components in normal and two rat models of ulcerative colitis (i.e., spleen deficiency with dampness retention-ulcerative colitis (SDDR-UC) rats and pure-ulcerative colitis (P-UC) rats). The pharmacokinetic parameters were significantly different among the three groups of rats. Overall, the absorption of the components was shown as follows: normal group > SDDR-UC group > P-UC group. The study could provide a scientific basis for further studies on pharmacokinetics and clinical differential application of SDDR-UC and P-UC patients.

## 1. Introduction

Ulcerative colitis (UC) is a chronic nonspecific inflammatory disease of colon and proctitis, and the lesions of UC mainly involved the colonic mucosa and submucosa, the main symptoms of which commonly include diarrhea, abdominal pain or discomfort, and even bloody stool. Because of its complex pathogenesis, lingering course, and many complications, UC has been listed as one of the modern refractory disorders by the World Health Organization (WHO) [1, 2]. Currently, there are several clinical types, such as initial, chronic recurrent, regular, persistent, and acute types [3]. However, the etiology and pathogenesis of this disease have not been fully clarified in modern medicine. Common drug therapies such as aminosalicylic acid preparation, glucocorticoid hormone, and immunomodulators are used to maintain and prevent the disease [4]. Although the treatment mentioned above is effective, it has serious side effects [5]. It is easy to relapse after drug withdrawal, causing significant damage to the patient's body with long-term maintenance treatment. Therefore, more and more therapies have begun to turn to traditional Chinese medicine (TCM). Some clinical studies [6] showed that TCM possessed significant advantages such as obvious curative efficacy, minor side effect, low recurrence rate, and low cost in the clinical treatment of colitis.

In the classic TCM book “Yellow Emperor's Inner Canon,” UC is described as “Chang Pi” and “Chi Wo,” and exogenous pathogens together with improper diet are two important links in the pathogenesis of UC [7]. According to the basic theory of TCM, spleen-stomach asthenia and maladjustment of transportation and transformation are the primary pathogenesis of UC [8]. On the basis of the classification standard formulated by the Spleen and Gastric Diseases Branch of China Association of Chinese Medicine [9], the classification of UC is as follows: (1) large intestine dampness-heat syndrome, (2) spleen deficiency with dampness retention syndrome, (3) cold and heat in complexity syndrome, (4) liver depression and spleen deficiency syndrome, (5) spleen-kidney yang deficiency syndrome, and (6) syndrome of yin-blood deficiency, among which spleen deficiency with dampness retention syndrome is the most common type in clinical concern to UC.

Shenling Baizhu San (SLBZS) is a representative prescription for invigorating spleen and resolving dampness. It comes from “Tai Ping Hui Min He Ji Ju Fang” (Chinese Song Dynasty), the first patent medicine standard edited by the government in the world. The formula consists of ten medical and edible herbs, namely, *Panax ginseng*, *Atractylodes macrocephala*, *Poria cocos*, *Nelumbo nucifera*, *Dioscorea opposita*, *Dolichos lablab*, *Coix lacryma-jobi*, *Platycodon grandiflorum*, *Amomum villosum*, and *Glycyrrhiza uralensis*. SLBZS has achieved sound effects in treating chronic UC for its efficacy of nourishing the spleen-stomach and benefiting Qi [10]. The primary chemical components of SLBZS mainly include triterpenoids (panaxadiol, ginsenoside Rg1, and platycodin D), sesquiterpenes (atractylenolide I, II, III), alkaloids (neferine and nuciferine), steroidal saponin (diosgenin), and flavone (isoliquiritigenin and liquiritigenin). These components all together show the effects of invigorating the spleen and supplementing Qi and draining dampness, as well as antidiarrheal and anti-inflammation effects. Panaxadiol alleviates inflammation by inhibiting immune inflammation [11]. Ginsenoside Rg1 has an anti-inflammatory effect by inhibiting inflammatory factors [12]. Atractylenolide I and III have the results of stimulating spleen activities and removing dampness, as well as antitumor, antibacterial, and anti-inflammation effects [13]. Pachymic acid possesses some functions such as anti-inflammatory, antioxidation, hypoglycemic, sedative, and hypnotic effects [14]. Neferine, the most abundant alkaloid in lotus seed, has protective cardiovascular, antithrombus, antioxidation, antitumor, and anti-inflammation impacts [15]. Nuciferine has a wide range of pharmacological activities, such as lowering blood lipid and hypoglycemia, as well as anti-inflammatory and anticancer activities [16]. The anti-inflammatory impact of diosgenin may be related to inhibiting leukocyte adhesion, migration, and inflammatory factors [17]. The saponins of *Platycodon grandiflorum* have anti-inflammation, antitumor, antiobesity, and other pharmacological effects [18]. Isoliquiritigenin may play an anti-inflammatory role by regulating the NF-*κ*B pathway [19].

In a word, the above studies showed that panaxadiol, ginsenoside Rg1, atractylenolide I, atractylenolide III, pachymic acid, neferine, nuciferine, diosgenin, platycodin D, and isoliquiritigenin can be characterized as the effective components of SLBZS in the treatment of UC. Several analytical assays have been reported for the determination of most of these representative ingredients by LC-MS and UHPLC method [20, 21]. However, to the best of our knowledge, there was no method established for quantitative analysis of ten components in biological fluids, let alone the publication describing the pharmacokinetic characteristics of SLBZS in treating SDDR-UC type at present.

Therefore, the present study aimed to develop a sensitive and reliable UHPLC-MS/MS method for the simultaneous determination of panaxadiol (PAN), ginsenoside Rg1 (Rg1), atractylenolide I (ATA-I), atractylenolide III (ATA-III), pachymic acid (PA), neferine (NEF), nuciferine (NUC), diosgenin (DG), platycodin D (PD), and isoliquiritigenin (ISL) to investigate the impact of their pharmacokinetic characteristics in normal, SDDR-UC, and P-UC rats. Furthermore, it was expected that the results of this study could provide a scientific basis for the clinical differential application of SDDR-UC and P-UC patients.

## 2. Materials and Methods

### 2.1. Materials and Reagents

**Figure 1:**
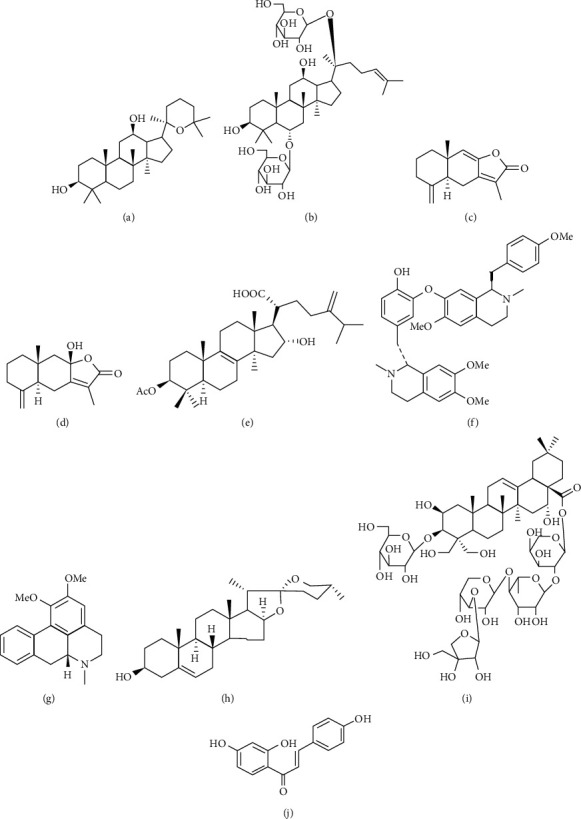
Chemical structures of ten analytes, panaxadiol (PAN), ginsenosideRg1 (Rg1), atractylenolide I (ATA-I), atractylenolide III (ATA-III), pachymic acid (PA), neferine (NEF), nuciferine (NUC), diosgenin (DG), platycodin D (PD), and isoliquiritigenin (ISL). (a) PAN, (b) Rg1, (c) ATA-I, (d) ATA-III, (e) PA, (f) NEF, (g) NUC, (h) DG, (i) PD, and (j) ISL.

Shenling Baizhu San was purchased from Tongrentong Pharmaceutical Co., Ltd., China. The reference standards of PAN, Rg1, ATA-I, ATA-III, PA, NEF, NUC, DG, PD, and ISL with purities of 98% were purchased from Chengdu Croma Biotechnology Co., Ltd. Puerarin (internal standard, IS) was obtained from Weikeqi Biological Technology Co., Ltd. The chemical structure of the above compounds is shown in Figure 1. Dextran sulfate sodium (DSS) was obtained from American MP Biomedicals. Formic acid was of chromatographic purity and was purchased from Dikma (Shanghai). HPLC-grade acetonitrile (ACN) and methanol (MeOH) were supplied by Merck Co. Ltd. (Merck, Germany) and used for HPLC analysis and plasma sample preparation. Deionized water was produced using a Milli-Q water purification system (Millipore, Bedford, MA, USA).

### 2.2. Instruments and LC-MS Conditions

The chromatographic separation was performed on a Shimadzu LC system (Kyoto, Japan) equipped with a pump (LC-30AD), autoinjector (SIL-30AC), online degasser (DGU-20A5), and column heater (CTO-30A5R). Chromatographic separation was accomplished on an ACE Excel 3 C18-AR column (100 mm × 2.1 mm, 3.0 *μ*m, Advanced Chromatography Technologies Ltd., Scotland). The mobile phase was composed of acetonitrile (solvent B) and water (solvent A, containing 0.1% formic acid) with a flow rate of 0.3 mL·min^−1^. The gradient elution program was set as follows: 2%–98% B at 0–5 min, 98%–2% B at 5–5.01 min, and 2% B at 5.01–7 min. The injection volume was 5 *μ*L with the temperature of the column maintained at 40°C.

**Figure 2:**
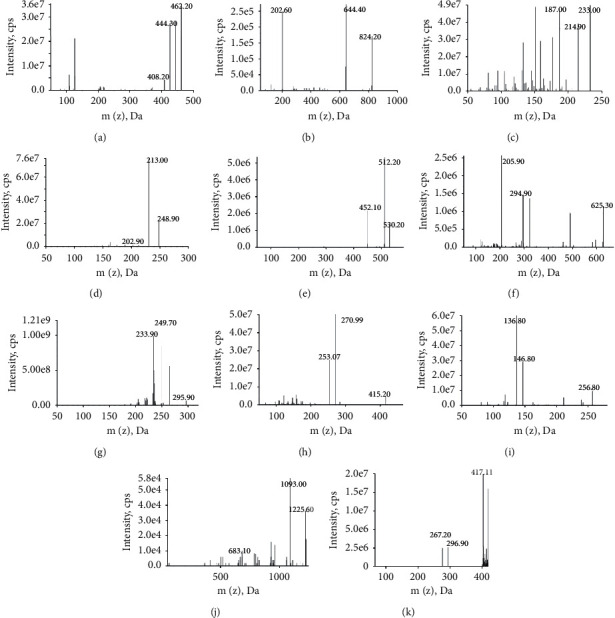
Full scan product ion spectra of (a) PAN, (b) Rg1, (c) ATA-I, (d) ATA-III, (e) PA, (f) NEF, (g) NUC, (h) DG, (i) PD, (j) ISL, and (k) IS.

A Triple Quad 5500 MS/MS system (AB SCIEX, Foster City, California, USA) was operated using an electrospray ionization (ESI) source in positive and multiple reactions monitoring (MRM) mode. The optimized ion spray voltage and source temperatures were 5500 V and 500°C. High-purity nitrogen generated by the nitrogen generator (99.999%, Peak Scientific Instruments Ltd., UK) was used as gas 1 (50 psi), gas 2 (45 psi), and curtain gas (35 psi). To optimize the MRM to each compound, the standard solution of each analyte was infused into the mass spectrometer in positive mode by the manual infusion using a syringe. The optimized MRM parameters, including collision energy (CE) and declustering potential (DP) of the ten analytes and IS, are listed in Table 1. The full scan product ion spectra of analytes and IS are provided in Figure 2. Analyst 1.6.2 software (AB SCIEX, USA) was used to control the equipment and acquire and analyze the data.

### 2.3. Preparation of Stock and Working Solution

The standard stock solutions of the ten analytes and IS were prepared by weighing appropriate amounts of PAN, Rg1, ATA-I, ATA-III, PA, NEF, NUC, DG, PD, ISL, and puerarin in the 10 mL volumetric mask, dissolving with methanol to the concentrations of 265, 379, 267, 327, 230, 186, 250, 298, 255, 237, and 208 *μ*g·mL^−1^, respectively. Then the stock solutions were further diluted with methanol to 1.47–1325.00, 2.1–1295.00, 1.48–1335.00, 1.81–1635.00, 1.27–1185.00, 1.03–930.00, 1.36–1225.00, 1.65–1490.00, 1.41–1275.00, 1.31–1187.00, and 1040 ng·mL^−1^, respectively, as the working solutions.

### 2.4. Preparation of Calibration Standard and Quality Control (QC) Samples

Calibration standard solutions were prepared by freshly spiking these working solutions into the blank rat plasma yielding the concentrations of 0.45, 4.02, 40.15, 100.38, 160.61, 267.67, and 401.50 ng·mL^−1^ for PAN, 0.64, 5.74, 57.42, 143.56, 229.69, 382.82, and 574.20 ng·mL^−1^ for Rg1, 0.44, 4.04, 40.45, 101.13, 161.81, 269.10, and 404.54 ng·mL^−1^ for ATA-I, 0.55, 4.95, 49.55, 123.86, 198.18, 321.21, and 495.45 ng·mL^−1^ for ATA-III, 0.38, 3.48, 34.84, 86.96, 139.39, 232.30, and 348.48 ng·mL^−1^ for PA, 0.31, 2.82, 28.20, 70.45, 112.73, 187.88, and 282.00 ng·mL^−1^ for NEF, 0.41, 3.71, 37.12, 92.80, 140.48, 247.55, and 371.21 ng·mL^−1^ for NUC, 0.5, 4.52, 45.20, 112.88, 180.66, 301.00, and 452.00 ng·mL^−1^ for DG, 0.43, 3.86, 38.6, 96.59, 154.55, 257.58, and 386.36 ng·mL^−1^ for PD, and 0.39, 3.59, 35.90, 89.77, 143.64, 239.39, and 359.00 ng·mL^−1^ for ISL. IS was set at the concentration of 104 ng·mL^−1^. Quality control (QC) samples were prepared for the intraday and interday accuracy and precision, extraction recovery, and stability study in the same way as calibration standard samples at three concentration levels of 0.80, 99.38, and 397.5 ng mL^−1^ for PAN, 1.14, 142.13, and 568.5 ng·mL^−1^ for Rg1, 0.80, 100.13, and 400.50 ng·mL^−1^ for ATA-I, 0.98, 122.63, and 490.50 ng·mL^−1^ for ATA-III, 0.69, 62.25, and 345.00 ng·mL^−1^ for PA, 0.31, 69.75, and 279.00 ng·mL^−1^ for NEF, 0.74, 69.75, and 225.00 ng·mL^−1^ for NUC, 0.89, 111.75, and 447.00 ng·mL^−1^ for DG, 0.77, 95.63, and 382.5 ng·mL^−1^ for PD,0.71, and 88.86 and 355.50 ng·mL^−1^ for ISL.

### 2.5. Sample Preparation

Frozen plasma samples were thawed and vortex-mixed. An aliquot of 30 *μ*L of ten analytes and 30 *μ*L IS solution were added to an Eppendorf tube (EP tube) and evaporated to dryness under the stream of nitrogen in a water bath at 40°C. Then an aliquot of 100 *μ*L thawed blank plasma was transferred into the above EP tube, and 400 *μ*L of acetonitrile was added and vortex-mixed for 1 min. After centrifugation at 13000 × g for 10 min, the supernatant was transferred to another EP tube and evaporated as described earlier. The residue was reconstituted with 100 *μ*L acetonitrile-0.1% formic acid aqueous solution (1 : 1, *v/v*), vortex-mixed for 1 min, and then centrifuged at 13000 × g for 10 min. The supernatant was injected into the UHPLC-MS/MS system for analysis.

### 2.6. Validation of the Method

#### 2.6.1. Specificity

The specificity was assessed by comparing the chromatograms of blank plasma obtained from rats with those of corresponding standard plasma sample spiked with PAN, Rg1, ATA-I, ATA-III, PA, NEF, NUC, DG, PD, ISL, and IS, as well as plasma samples collected at 0.5 h after oral administration of SLBZS.

#### 2.6.2. Linearity

The calibration curves were constructed by plotting the peak area ratios (analyte/IS) versus nominal concentrations using the weighted (1/*x*^2^) least-square linear regression method. The LLOQ was defined as the lowest concentration on the calibration curve.

#### 2.6.3. Extraction Recovery (Absolute Recovery) and Matrix Effect

The extraction recovery was evaluated by comparing the mean peak areas of the QC samples spiked before protein precipitation with those spiked after protein extraction. The absolute matrix effect was evaluated via comparing the mean peak areas of the QC samples spiked after pretreatment with those of the pure solution. The relative matrix effect was assessed by calculating the coefficient of variation CV (%) between the mean peak areas of the QC samples spiked after pretreatment with those of the pure solution. Five samples of the low, medium, and high concentrations of QC samples were processed in parallel.

#### 2.6.4. Precision and Accuracy

The intraday precision and accuracy were calculated by continuously measuring a batch of QC samples on the same day. The interday precision and accuracy were tested in three batches on different consecutive days. Precision was expressed by the relative standard deviation (R.S.D, %), while accuracy (%) was evaluated by the percentage difference between the mean measured concentrations and the spiked concentrations.

#### 2.6.5. Stability

The stability tests of the ten analytes were assessed by comparing measured QC samples' results with those of freshly prepared samples under different conditions. The postpreparation stability was carried out by detecting the samples in the autosampler (4°C) for 12 h; the short- and long-term stabilities were evaluated by analyzing samples at room temperature for 4 h and in the freezer (−20°C) for 30 days, respectively; the freeze-thaw stability was assessed by determining samples undergoing three freeze-thaw cycles (from −20°C to room temperature)

### 2.7. Pharmacokinetic Study

Specially pathogen-free Sprague-Dawley rats (male, weighing 220 ± 20 g) were purchased from Hunan Slac Laboratory Animal Co., LTD. (Hunan, China, certificate no. SCXK (Xiang) 2019–0004) and acclimated in Exhaust Ventilated Closed-System Cage Rack (EVC) for at least a week with environmentally controlled quarters (22 ± 2°C and 12/12 h light/dark cycle) and free access to standard chow and water. Animal welfare and experimental procedures were strictly in accordance with the guide for the care and use of laboratory animals by the Animal Ethics Committee of the Jiangxi University of TCM. After one week of acclimatization, the rats were randomly divided into three groups with 8 rats in each group: the normal control (one rat died in the normal control group after collecting blood from retinal vein plexus of rats), SDDR-UC model group, and P-UC model group. The SDDR-UC and P-UC rat models were established according to the previous study [22]. Briefly, except for the normal group, the SDDR-UC model group was established by the combination of disease and syndrome, including diet and environment intervention for 48 days and administrated intragastrically with 5% DSS (0.2 g·kg^−1^·d^−1^) for 8 days, and the P-UC model group was only with 5% DSS. The symptom score standard was established according to the macroscopic signs (such as feces, diet, drinking water, weight, skin, hair, mental state, etc.). The model of spleen deficiency and dampness (SDDR) was developed successfully when the score was more than four. The disease activity index (DAI) was used to evaluate UC, and hematoxylin-eosin (HE) staining was used to observe the pathological changes of the colon in different groups [23].

After modeling, each group of rats was gavaged 0.945 g·kg^−1^ SLBZS, and small amounts of diethyl ether anesthetized the rats. Subsequently, approximately 0.2 mL blood was collected from retinal vein plexus of rats into heparinized tubes at predetermined time points (0, 0.083, 0.25, 0.5, 1, 1.5, 2, 4, 6, 8, 12, and 24 h) after drug administration. Then, more than 100 *μ*L plasma was obtained by centrifugation at 13000 × g for 10 min and stored at −20°C until analysis.

### 2.8. Pharmacokinetic Study and Data Analysis

The pharmacokinetic parameters, that is, maximum plasma concentration (*C*_max_), corresponding time (*t*_max_), half-life (*t*_1/2_), area under the plasma concentration-time curve (AUC), plasma clearance (CL), apparent volume of distribution (Vz), and mean residence time (MRT) were performed on each individual set using the software of WinNonlin (Version 4.1, Pharsight Corp, Mountain View, CA, USA) by the noncompartmental model. Data are presented as Mean ± SD. Student's *t*-test was used to compare the pharmacokinetic data, and the statistically significant difference was set at a value of *P* < 0.05 (GraphPad Prism software package, Version 6.0).

## 3. Results

### 3.1. Method Validation

#### 3.1.1. Specificity

The retention times of PAN, Rg1, ATA-I, ATA-III, PA, NEF, NUC, DG, PD, ISL, and Pur (IS) were 9.24, 4.40, 6.45, 5.97, 7.91, 4.22, 5.05, 9.48, 4.66, 5.45, and 3.70 min, respectively. No significant interference from endogenous substances was observed at the retention time of the analytes and IS.

**Figure 3:**
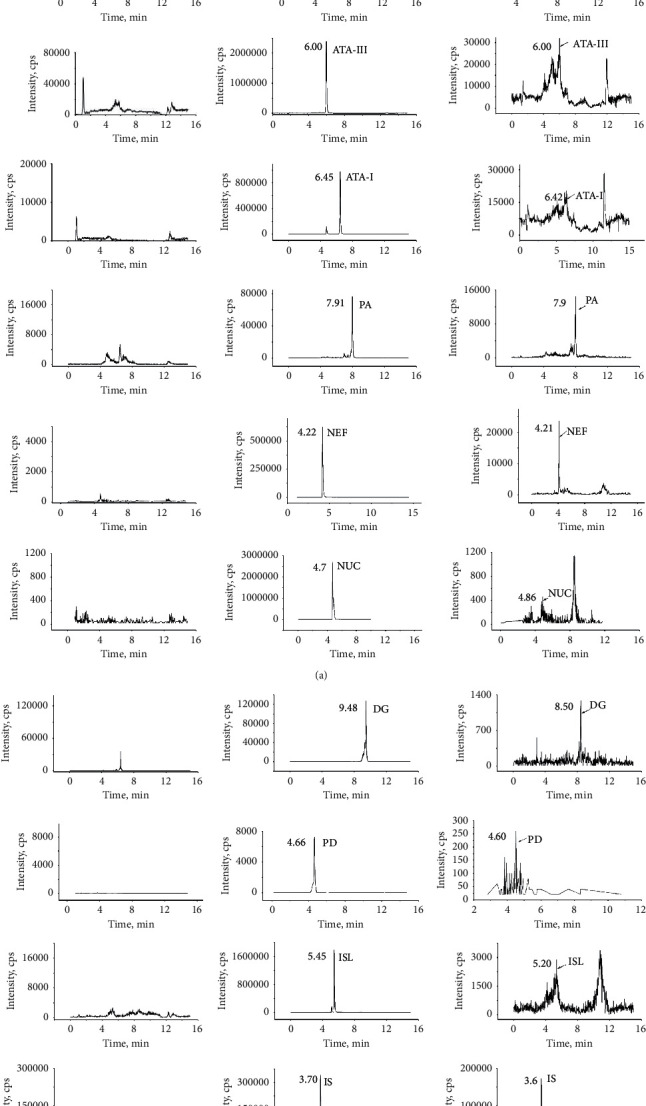
Representative MRM spectrum of each component: (a) blank plasma; (b) blank plasma spiked with analytes and IS; (c) rat plasma samples obtained at 0.5 h after oral administration of SLBZS.

Figure 3 shows the representative chromatograms of blank plasma, corresponding samples spiked with IS/analyte, and rat plasma samples collected at 0.5 h after administration.

#### 3.1.2. Linearity

The calibration curve of the analytes was established with more than six points of standard solution. The curves exhibited good linearity with correlation coefficients greater than 0.999, and the regression equations, linear ranges, correlation coefficients, and LLOQ for the ten analytes are shown in Table 2.

#### 3.1.3. Extract Recovery (Absolute Recovery) and Matrix Effect

The absolute recoveries were all more than 50% at each QC level, which satisfied the quantitative requirements of biological samples. Concerning the matrix effect, no significant matrix effects were observed for the ten analytes and IS. In other words, the responses of the ten analytes in the matrix were consistent with that in the standard solution. These results are shown in Table 3.

#### 3.1.4. Precision and Accuracy (Relative Recovery)

The intraday precision and interday precision (R.S.D, %) of the ten analytes were all less than 15%, and the accuracy (relative recovery) was above 85%, which indicated that the established method was accurate and precise (also shown in Table 3).

#### 3.1.5. Stability

As shown in Table 4, all analytes remained generally stable in plasma under a variety of storage and process conditions: for 4 h at room temperature, 30 days when stored at −20°C for three freeze-thaw cycles, and 12 h when stored in autosampler at 4°C.

### 3.2. Pharmacokinetic Study

**Figure 4:**
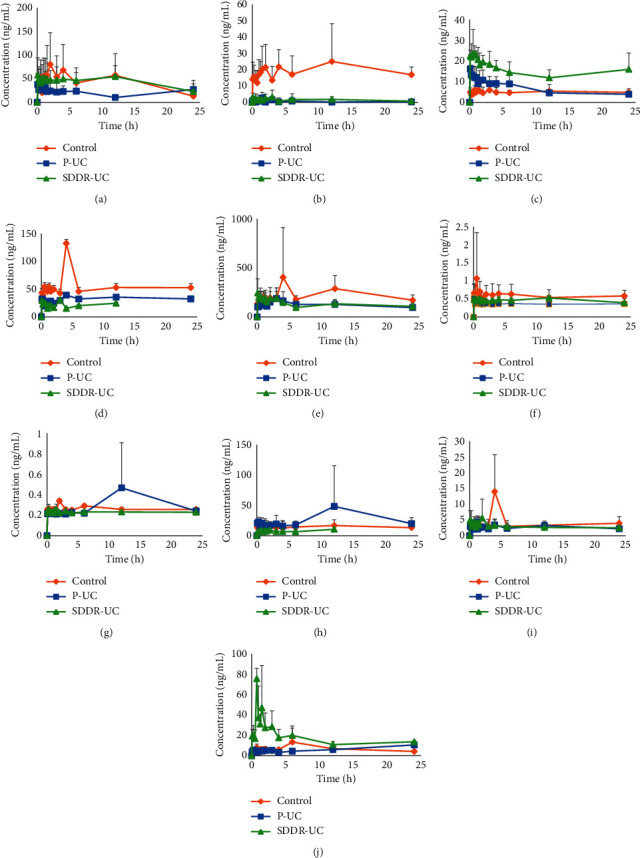
Mean concentration-time curves in rat plasma after oral administration of SLBZS (Mean ± SD, *n* = 7 in normal and *n* = 8 in SDDR-UC and P-UC group). (a) PAN, (b) Rg1, (c) ATA-I, (d) ATA-III, (e) PA, (f) NEF, (g) NUC, (h) DG, (i) PD, and (j) ISL.

**Figure 5:**
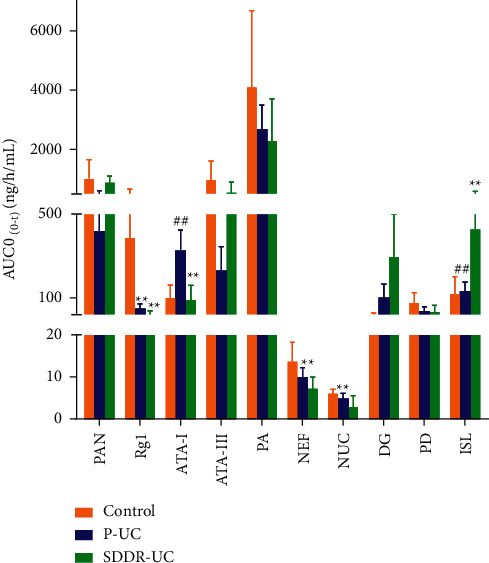
AUC_(0-*t*)_ in rat plasma after oral administration of SLBZS (Mean ± SD, *n* = 7 in normal and *n* = 8 in SDDR-UC and P-UC group).

The UHPLC-MS/MS method was successfully applied to a comparative pharmacokinetic study of ten compounds in normal, SDDR-UC, and P-UC rats after oral administration of SLBZS.  Figure 4 shows the mean plasma concentration-time profiles of the ten analytes in rat plasma after oral administration of SLBZS. AUC_(0-*t*)_ of the ten analytes in rat plasma after oral administration of SLBZS are shown in Figure 5. Pharmacokinetic parameters of *C*_max_, *T*_max_, *t*_1/2_, AUC_(0-*t*)_, AUC_(0-∞)_, CL, Vz, MRT_(0-*t*)_, and MRT_(0-∞)_ for the ten analytes detected after administration of SLBZS are shown in Table 5.

*T*_max_ of ATA-I in the SDDR-UC group was significantly shortened compared to that of the normal group (*P* < 0.05). At the same time, *t*_1/2_, AUC_(0-*t*)_, and MRT_(0-∞)_ were significantly prolonged, indicating that SDDR-UC increased the absorption of ATA-I but slowed down its elimination. *t*_1/2_ and CL of ATA-III in the SDDR-UC group were significantly increased (*P* < 0.05), implying that SDDR-UC slowed down their elimination. *C*_max_ and AUC_(0-*t*)_ of Rg1 in SDDR-UC group were significantly decreased (*P* < 0.05), indicating that SDDR-UC reduced the absorption of Rg1. Compared with the normal group, the pharmacokinetic parameters of Rg1 and ATA-I in P-UC group were similar to those in SDDR-UC group (*P* < 0.05). *t*_1/2_ of NUC in the P-UC group was relatively high. Still, AUC_(0-*t*)_ and MRT_(0-∞)_ were relatively short (*P* < 0.05), which indicated decreasing the absorption of NUC and accelerating its elimination. In addition, it was found that AUC (ATA-I, ISL) and *t*_1/2_ (ATA-I) of SDDR-UC rats were higher (*PP* < 0.05) than those of the P-UC group, indicating that the systemic exposure of SLBZS in SDDR-UC rats was significantly increased to a certain extent.

It is worth noting that some plasma concentration data fluctuate significantly, which is due to individual differences. Concomitantly, there was no apparent double peaks phenomenon of the analytes after administration of SLBZS.

## 4. Discussion

The mobile phase selection is essential for improving peak shape, obtaining proper retention, increasing the signal response of analytes, and shortening run time. In our analysis, we compared the acetonitrile-0.1% formic acid and acetonitrile-10 mmol acetic acid systems. It was found that acetonitrile-0.1% formic acid produces better peak shapes and lower background noise than acetonitrile-10 mmol acetic acid. Besides, the acetonitrile-0.1% formic acid in water could attain a higher response of all the analytes, which was finally adopted. In addition, the extraction solvents in pretreating plasma samples were investigated, including acetonitrile, methanol, and ethyl acetate. By comparing impurities and extraction recovery interference, it was found that acetonitrile was the best solvent for protein precipitation.

To optimize MS/MS parameters, individual standard solution was directly infused into the mass spectrometer in both positive and negative modes. The observed mass spectral response and stability of ten analytes and IS were higher in positive mode than in negative ion mode. The MS/MS product ion spectra of the analytes and IS are shown in Figure 2. On the basis of that, to get the richest mass spectral abundance of precursor and productions, the parameters for DP and CE were further optimized (Table 1).

This is the first systematic study determining the pharmacokinetic behaviors of PAN, Rg1, ATA-I, ATA-III, PA, NEF, NUC, DG, PD, and ISL as main bioactive components of SLBZS in normal and two models of rats. Results of current study showed that the two model rats' pharmacokinetic behaviors differed compared to the normal group, especially in *t*_1/2_, AUC_(0-*t*)_, and MRT_(0-∞)_. AUC_(0-*t*)_ of most components in SLBZS in the two models group was higher than that in the normal group (see Table 5 and Figure 5), which suggested that the absorption was decreased in the two models of rats.

The liver plays an essential role in drug metabolism and the cytochrome P_450_ (CYP) is the main enzyme system involved in drug metabolism. Recent studies have shown that inflammation increases CYP3A4 in rat liver tissue and enhances enzyme activity [24]. The increase of metabolic enzyme activity might be related to the change of drug disposition. Therefore, the increase of metabolic enzyme activity may lead to a decrease in the absorption of the main active ingredients in SLBZS.

Additionally, the absorption of SLBZS may also be involved in intestinal changes. It has been reported that the pathogenesis of UC is related to intestinal dysfunction [25]. In an inflammatory state, inflammatory factors can change liver and gut-related transporter expression (such as P-glycoprotein (P-gp)), the top sodium-dependent bile salt transporter (ASBT), and bile salt excretion pump (BSEP) proteins under the inflammatory state. It has been reported that UC may increase the content of P-gp in the intestine [26]. Although P-gp is not directly involved in the metabolism of drugs, it still impacts the metabolism of drugs in the intestine. It can discharge drugs from intestinal epithelial cells into the adjacent lacuna, thus accelerating the elimination of drugs from the intestinal mucosa and reducing the efficacy. Therefore, the increased expression of P-gp may be involved in the decreased absorption process of SLBZS. Studies have shown that the expression of ASBT and BSEP proteins is downregulated. The homeostasis of enterohepatic circulation is broken, so the drugs affected by which cannot be reabsorbed or reabsorbed slowly, resulting in the shortening of the action time and lowering the efficacy of drugs [27, 28]. Our study shows the decrease of ginsenoside Rg1 absorption after modeling maybe because Rg1 undergoes enterohepatic circulation, and inflammatory factors affect the enterohepatic circulation process, resulting in the slow absorption of Rg1 in the intestine [29]. So studies have shown that Rg1 is easy to be degraded by enzymes in intestinal bacteria. The oral absorption is deficient, and the elimination in the blood is accelerated so that the absorption of Rg1 is reduced [30]. To our knowledge, as for most alkaloids in Chinese Materia Medica, it is generally known that the poor transmembrane transport and low absorption in the small intestine lead to their low bioavailability. For example, the concentration of NUC in this paper is deficient, for it is difficult to enter the blood circulation through intestinal epithelial cells in large quantities. Gu et al. [31] also found that nuciferine had poor absorption from the gastrointestinal tract in rats, which is consistent with our results. Another study has also reported that the concentration of NUC is highest in the liver and kidney and lower in blood, which may be the reason for the decrease in NUC absorption [32].

The pharmacokinetic parameters of SLBZS in the two models also showed significant differences between them. In general, the absorption and retention time of SLBZS in the SDDR-UC group were higher than those in the P-UC group. Studies have shown that the mucosal damage and intestinal permeability of SDDR-UC increased [33], but no study reported their severity and difference of intestinal damage with the P-UC model. In our previous study [23], by detecting the contents of inducible nitric oxide synthase (iNOS), procalcitonin (PCT), C-reactionprotein (CRP), and myeloperoxidase (MPO) in the serum of rats, it was found that the contents of iNOS, PCT, CRP, and MPO in the two model rats were significantly increased, which indicated that the inflammation level of model rats was higher than that of normal rats; and there was no significant difference between the two model groups. However, after administration of SLBZS, the contents of PCT and CRP were significantly decreased in serum of rats with SDDR-UC, indicating that SLBZS had a better therapeutic effect on the rats with SDDR-UC than on the rats with P-UC. Spleen deficiency and dampness are the keys to the onset of SDDR-UC, so the main treatment principle is to invigorate the spleen and infiltrate dampness. SLBZS is the representative prescription for invigorating spleen and resolving dampness, so it has a better treatment effect on SDDR-UC type. This theoretical basis has also been confirmed in our previous pharmacodynamic results. In this paper, further research is carried out to elucidate the difference of SLBZS in treating SDDR-UC type and P-UC rats from the perspective of pharmacokinetics.

## 5. Conclusion

In the present study, a selective and sensitive UHPLC-MS/MS method was developed and successfully applied to the simultaneous determination of ten significant components (PAN, Rg1, ATA-I, ATA-III, PA, NEF, NUC, DG, PD, and ISL) in the plasma of normal and two models of rats. The results demonstrated significant differences in some pharmacokinetic parameters in normal, SDDR-UC, and P-UC groups. Overall, the absorption and action time of SLBZS in the three groups were as follows: normal group > SDDR-UC group > P-UC group. The results could be helpful to facilitate the further research of the mechanism of SLBZS and provide useful information for the clinical differential application of SDDR-UC and P-UC patients. However, the specific mechanism of SLBZS needs to be further studied from the small molecular metabolites, the genetic level, and the changes in the intestinal flora.

### 5.1. Highlights

- A UHPLC-MS/MS method was developed and fully validated for the simultaneous determination of five different types of compounds in rat plasma for the first time.
- This method was successfully applied to the simultaneous determination of ten active components in normal and two models of ulcerative colitis (SDDR-UC and P-UC) rats after oral administration of SLBZS.
- The pharmacokinetic parameters were significantly different among the three groups of rats. Overall, the absorption of the components was shown as follows: normal group > SDDR-UC group > P-UC group.

## Figures and Tables

**Table 1:** Optimized precursor/production pairs and MRM parameters for the analytes and IS.

Analyte	Precursor ion	Product ion quantifier/qualifier	DP	CE
PAN	462.20	444.30/408.20	36	17
Rg1	824.20	644.40/202.60	19	50
ATA-I	233.00	187.00/214.90	107	23
ATA-III	248.90	213.00/202.90	50	14
PA	530.20	512.20/452.10	54	17
NEF	625.30	205.90/294.90	94	44
NUC	295.90	233.90/249.70	78	29
DG	415.20	270.99/253.07	74	28
PD	1225.60	1093.00/683.10	34	20
ISL	256.80	136.80/146.80	81	28
Puerarin (IS)	417.11	296.90/267.20	150	25

**Table 2:** Linearity of each component (*n* = 5).

Analyte	Calibration curves	Correlation coefficient (r)	Linear range (ng·mL^−1^)	LLOQ (ng·mL^−1^)
PAN	*Y* = 0.00102*X* + 6.1385*e* − 4	0.9990	0.44∼397.50	0.44
Rg1	*Y* = 2.525*e* − 5*X* + 9.435*e* − 5	0.9990	0.63∼388.50	0.63
ATA-I	*Y* = 0.00290*X* − 0.00119	0.9990	0.44∼400.50	0.44
ATA-III	*Y* = 0.00439*X* + 0.22385	0.9990	0.54∼490.50	0.54
PA	*Y* = 7.6916*e *− 5*X* + 0.00537	0.9995	0.38∼355.50	0.38
NEF	*Y* = 0.00133*X* + 0.00776	0.9994	0.31∼279.00	0.31
NUC	*Y* = 0.02406*X* − 0.00469	0.9991	0.41∼367.50	0.41
DG	*Y* = 2.563*e* − 4*X* + 0.00142	0.9994	0.50∼447.00	0.50
PD	*Y* = 3.5807*e* − 5*X* + 2.740*e* − 5	0.9990	0.42∼382.50	0.42
ISL	*Y* = 0.00220*X* − 2.625*e* − 4	0.9992	0.39∼356.10	0.39

**Table 3:** Extract recovery, matrix effect, precision, and accuracy data of the analytes in rat plasma (*n* = 5).

Analyte	Concentration (ng·mL^−1^)	Absolute recovery (%)	Absolute matrix effect (%)	Relative matrix effect (%)	Precision% (RSD)		Accuracy% (Mean ± SD)	
					Intraday	Interday	Intraday	Interday
PAN	0.80	93.68 ± 8.33	52.84 ± 2.44	6.59	12.14	12.14	82.34 ± 5.42	103.54 ± 13.47
	99.38	93.06 ± 8.24	56.56 ± 2.35	7.81	7.60	8.20	101.60 ± 8.22	99.97 ± 6.012
	397.50	92.74 ± 4.43	46.34 ± 9.01	9.86	6.59	6.47	104.46 ± 3.79	99.99 ± 17.16

Rg1	1.14	66.92 ± 7.47	102.59 ± 1.56	11.58	10.41	9.95	98.3 ± 8.08	99.75 ± 2.79
	142.13	65.06 ± 5.40	93.92 ± 5.68	13.05	13.60	10.41	114.75 ± 1.30	90.02 ± 2.20
	568.50	53.71 ± 1.14	46.65 ± 12.63	11.49	11.91	13.60	91.57 ± 2.80	92.01 ± 3.50

ATA-I	0.80	56.89 ± 7.00	75.07 ± 13.32	8.17	4.47	1.89	119.38 ± 7.10	93.75 ± 1.08
	100.13	65.26 ± 1.00	70.17 ± 4.24	7.82	1.85	3.78	90.83 ± 4.47	100.02 ± 2.23
	400.50	77.85 ± 1.44	67.40 ± 3.57	5.88	13.30	12.47	102.29 ± 11.23	99.03 ± 5.96

ATA-III	0.98	66.92 ± 7.47	48.99 ± 3.94	5.54	6.69	8.09	119.25 ± 10.37	101.10 ± 6.55
	122.63	65.06 ± 5.4	93.56 ± 4.43	9.49	8.30	5.90	96.99 ± 10.89	93.58 ± 12.93
	490.50	98.81 ± 5.38	82.58 ± 9.47	11.67	11.17	10.84	100.61 ± 6.42	95.84 ± 7.01

PA	0.69	79.86 ± 6.63	54.98 ± 12.72	10.73	1.89	9.93	91.11 ± 10	103.41 ± 3.17
	62.25	85.95 ± 12.08	74.95 ± 4.71	10.27	13.89	14.55	98.54 ± 3.25	101.02 ± 2.32
	345.00	99.46 ± 1.89	71.18 ± 9.29	5.84	8.30	9.48	103.59 ± 2.21	94.22 ± 4.09

NEF	0.31	55.13 ± 4.65	41.51 ± 3.49	9.58	6.24	6.10	102.55 ± 2.05	110.41 ± 2.62
	69.75	53.67 ± 1.46	34.17 ± 1.85	10.97	4.77	3.57	109.22 ± 2.13	98.37 ± 4.14
	279.00	78.85 ± 2.97	74.39 ± 1.43	9.71	7.42	10.50	95.57 ± 2.48	94.22 ± 3.09

NUC	0.74	65.79 ± 3.49	58.7 ± 7.32	7.94	11.16	10.13	111.07 ± 1.61	99.77 ± 3.38
	69.75	57.76 ± 10.04	73.32 ± 3.94	9.38	10.11	9.08	111.73 ± 5.80	101.44 ± 5.30
	225.00	65.79 ± 6.17	56.92 ± 5.47	13.86	5.30	10.49	96.17 ± 5.32	99.25 ± 9.69

DG	0.89	73.97 ± 2.81	77.47 ± 8.16	11.71	14.55	7.23	106.74 ± 1.36	102.68 ± 3.72
	111.75	60.05 ± 8.30	127.46 ± 9.34	5.81	8.86	10.44	100.01 ± 1.5	100.89 ± 3.7
	447.00	55.79 ± 1.04	75.49 ± 9.74	8.43	13.8	12.31	104.28 ± 1.78	94.98 ± 1.87

PD	0.77	94.58 ± 5.68	58.48 ± 11.23	10.73	6.01	6.84	99.95 ± 6.42	107.93 ± 3.31
	95.63	84.78 ± 12.69	135.38 ± 15.43	5.84	12.69	12.67	100.20 ± 10.89	108.66 ± 1.76
	382.50	61.01 ± 5.75	115.65 ± 5.36	10.27	12.15	12.91	109.25 ± 10.37	105.23 ± 1.01

ISL	0.71	68.86 ± 8.93	54.78 ± 5.27	12.50	1.61	7.064	96.40 ± 7.149	100.76 ± 9.77
	88.86	65.71 ± 5.00	89.26 ± 6.30	13.44	7.60	10.32	98.27 ± 5.47	95.410 ± 3.09
	355.50	64.84 ± 4.88	85.51 ± 8.51	14.39	12.96	12.0	100.72 ± 3.7	93.01 ± 12.45

IS	104	94.51 ± 4.29	99.84 ± 7.96	—	—	—	—	—

**Table 4:** Stability of the components in rat plasma under a variety of storage and process conditions (*n* = 5).

Analyte	Concentration (ng·mL^−1^)	RSD%			
		Short-term stability (room temperature, 4 h)	Autosampler stability (4°C, 12 h)	Freeze-thaw cycles (three freeze-thaw cycles)	Long-term stability (−20°C, 30 d)
PAN	0.80	6.85	4.54	10.71	12.81
	99.38	6.24	7.61	10.77	8.94
	397.50	6.76	3.57	11.82	6.43

Rg1	1.14	3.38	3.38	14.47	10.79
	142.13	3.41	3.41	9.60	7.60
	568.50	10.88	8.21	7.19	8.95

ATA-I	0.80	8.86	8.86	11.17	6.05
	100.13	11.79	11.79	6.24	8.53
	400.50	8.59	8.59	9.48	6.56

ATA-III	0.98	10.49	10.49	11.56	2.56
	122.63	7.59	7.59	9.19	7.01
	490.50	3.12	3.12	9.35	9.19

PA	0.69	6.81	7.55	10.06	10.06
	62.25	6.34	3.92	13.50	13.50
	345.00	8.88	2.26	5.01	5.01

NEF	0.31	3.85	6.63	2.95	7.93
	69.75	5.27	7.61	9.26	13.41
	279.00	2.16	5.15	7.86	14.69

NUC	0.74	4.11	14.46	12.64	13.16
	69.75	3.41	8.23	12.59	11.06
	225.00	4.01	6.36	6.27	11.19

DG	0.89	12.11	4.39	3.99	8.52
	111.75	9.85	3.12	13.42	10.94
	447.00	7.59	1.82	9.86	9.65

PD	0.77	11.69	6.16	11.97	15.38
	95.63	5.01	2.99	5.27	11.74
	382.50	3.27	5.84	7.83	10.07

ISL	0.71	5.72	7.13	5.72	11.80
	88.86	1.35	1.54	1.35	13.57
	355.50	2.21	6.22	2.21	12.65

**Table 5:** Mean pharmacokinetic parameters of active ingredients of SLBZP in normal and two model rats (Mean ± SD, *n* = 7 in normal and *n* = 8 in SDDR-UC and P-UC group).

Analyte	Group	*C*_max_ (ng·mL^−1^)	*T*_max_ (h)	*t*_1/2_ (h)	AUC_(0-t)_ (ng·h·mL^−1^)	AUC_(0-∞)_ (ng·h·mL^−1^)	CL (mL·h·kg^−1^)	Vz (mL·kg^−1^)	MRT_(0-t)_ (h)	MRT_(0-∞)_ (h)
PAN	Control	161.08 ± 124.22	3.18 ± 3.75	1.08 ± 0.64	897.55 ± 666.85	931.26 ± 667.45	5.37 ± 3.28	7.59 ± 4.28	7.11 ± 3.76	7.57 ± 3.81
	SDDR-UC	106.82 ± 22.17	0.74 ± 0.47	1.82 ± 0.74	929.83 ± 172.24	983.53 ± 159.78	4.24 ± 0.6	11.1 ± 4.8	8.78 ± 2.23	9.61 ± 2.04
	P-UC	52.93 ± 12.26^*∗*^	1.13 ± 0.92	1.34 ± 1.05	367.82 ± 222.72	406.79 ± 241.38	10.29 ± 7.1	20.26 ± 19.14	8.93 ± 5.33	9.91 ± 5.27

Rg1	Control	59.14 ± 47.02	4.64 ± 3.39	0.77 ± 0.46	393.38 ± 264.89	416.64 ± 265.22	6.71 ± 1.93	14.25 ± 10.68	8.41 ± 4.49	9.02 ± 4.67
	SDDR-UC	8.02 ± 2.06^*∗*^	2.38 ± 2.09	0.67 ± 0.45	53.63 ± 17.95^*∗∗*^	55.78 ± 17.3^*∗∗*^	74.46 ± 42.94	69.92 ± 48.68	7.77 ± 4.76	8.18 ± 5.08
	P-UC	4.5 ± 1.62^*∗∗*^	1.1 ± 1.03	0.39 ± 0.27	22.54 ± 15.52^*∗∗*^	27.02 ± 15^*∗∗*^	132.62 ± 71.81^*∗∗*^	76.87 ± 34.24	8.47 ± 6.88	9.93 ± 6.89

ATA-I	Control	14.81 ± 16.3	3.64 ± 3.58	1.17 ± 0.42	105.09 ± 55.63	113.37 ± 56.79	174.76 ± 284.12	223.16 ± 289.35	8.56 ± 4.46	9.38 ± 4.54
	SDDR-UC	26.97 ± 4.42	0.35 ± 0.3^*∗*^	25.5 ± 9.35^*∗∗*^	330.81 ± 93.23^*∗∗*^	829.17 ± 150.77^*∗∗*^	8.4 ± 1.6	303.92 ± 113.96	9.18 ± 2.17	38.44 ± 11.05^*∗∗*^
	P-UC	16.16 ± 7.09	0.11 ± 0.06^*∗*^	1.93 ± 1.32^##^	96.09 ± 63.88^##^	113.83 ± 66.19^##^	128.62 ± 159.26	223.47 ± 152.6	6.15 ± 4.31	7.64 ± 4.55^##^

ATA-III	Control	155.12 ± 170.33	3.82 ± 3.56	0.55 ± 0.22	1010.09 ± 596.3	1050.66 ± 598.91	64.02 ± 80.94	36.43 ± 37.03	8.79 ± 4.94	9.19 ± 4.99
	SDDR-UC	43.45 ± 10.1	2.58 ± 3.95	1.68 ± 1.08^*∗*^	235.28 ± 109.21	283.36 ± 111.31^*∗∗*^	103.65 ± 50.43^*∗∗*^	231.28 ± 141.63	6.14 ± 3.75	7.54 ± 3.78
	P-UC	52.15 ± 10.74	3.01 ± 3.91	0.78 ± 0.38	583.08 ± 315.6	615.63 ± 315.18	82.34 ± 97.68	105.26 ± 163.44	9.03 ± 4.57	9.64 ± 4.49

PA	Control	312.25 ± 94.35	4.29 ± 3.49	1.07 ± 0.85	4139.68 ± 2544.72	4388.37 ± 2664.79	2.48 ± 0.61	4.75 ± 2.66	8.69 ± 4.68	9.35 ± 4.79
	SDDR-UC	292.12 ± 123.48	0.74 ± 0.81^*∗*^	3.45 ± 1.8^*∗*^	2713.48 ± 779.08	3227 ± 971.31	4.93 ± 2.24	21.46 ± 8.42	8.93 ± 2.52	11.59 ± 3.95
	P-UC	251.2 ± 90.14	1.5 ± 0.83	1.98 ± 1.31	2319.51 ± 1382.55	2572.66 ± 1469.32	12.26 ± 14.82	20.11 ± 13.19	8.23 ± 4.27	9.57 ± 4.54

NEF	Control	1.33 ± 1.09	1.3 ± 1.94	1.02 ± 0.19	14 ± 4.24	14.86 ± 4.44	121.99 ± 30.82	179.57 ± 60.51	11.81 ± 0.57	12.6 ± 0.74
	SDDR-UC	0.65 ± 0.07	3.82 ± 5.18	2.45 ± 1.42^*∗*^	10.19 ± 2.04	11.63 ± 2.34	152.39 ± 41.13	497.98 ± 238.53	10.9 ± 2.03	12.81 ± 2.76
	P-UC	0.63 ± 0.35	1.25 ± 1.98	2.36 ± 0.62	7.55 ± 3.36^*∗∗*^	8.76 ± 3.56^*∗∗*^	240.36 ± 157.89	712.45 ± 228.39	10.46 ± 4.63	12.4 ± 4.75

NUC	Control	0.37 ± 0.19	9.15 ± 7.83	1.88 ± 0.81	6.33 ± 0.74	7.01 ± 0.58	29.17 ± 2.32	80.56 ± 37.25	11.88 ± 0.6	13.39 ± 1.12
	SDDR-UC	0.29 ± 0.06	5.18 ± 7.9	2.6 ± 1.27	5.13 ± 0.97	5.98 ± 0.77	34.68 ± 5.68	133.56 ± 75.64	11.18 ± 2.17	13.39 ± 1.83
	P-UC	0.36 ± 0.25	8.96 ± 10.25	5.7 ± 3.19^*∗*#^	3.15 ± 2.43^*∗∗*^	6.72 ± 6.04	66.23 ± 55.8	418.15 ± 293.11	6.86 ± 5.32^*∗*^	13.66 ± 6.41

DG	Control	23.83 ± 5.32	3.05 ± 3.83	2.51 ± 0.83	259.36 ± 200.48	309.63 ± 231.1	0.86 ± 0.94	2.9 ± 3.35	8.77 ± 4.78	10.75 ± 4.91
	SDDR-UC	14.67 ± 2.52	6.18 ± 8.21	1.13 ± 0.56^*∗∗*^	106.53 ± 59.84	123.81 ± 69.4	1.38 ± 0.91	1.65 ± 0.57	7.19 ± 4.03	8.18 ± 4.44
	P-UC	49.54 ± 48.87	2.18 ± 4.05	0.88 ± 0.51^*∗∗*^	300.44 ± 204.52	321.14 ± 218.5	1.28 ± 2.09	0.92 ± 0.92	8.86 ± 4.77	9.46 ± 4.91

PD	Control	5.63 ± 1.29	3.17 ± 1.8	5.25 ± 3.35	71.99 ± 47.81	103.47 ± 62.5	1403.02 ± 832.03	10564.73 ± 4303.49	9.23 ± 5.51	13.96 ± 8.04
	SDDR-UC	6.44 ± 1.49	1.11 ± 0.52	2.42 ± 1.29	52.57 ± 31.06	61.89 ± 29.36	2598.65 ± 1218.3	8646.19 ± 4540.35	9.4 ± 4.34	11.62 ± 5
	P-UC	5.23 ± 0.9	1.49 ± 1.63	2.63 ± 1.79	39.16 ± 25.57	49.35 ± 28.22	3321.16 ± 2712.77	11129.86 ± 4568.62	7.15 ± 4.35	9.42 ± 5.26

ISL	Control	13.98 ± 10.03	3.94 ± 4.01	1.05 ± 0.48	125.15 ± 76.25	131.71 ± 76.69	497.73 ± 102.18	748.28 ± 252.22	7.83 ± 4.3	8.44 ± 4.24
	SDDR-UC	110.61 ± 94.62^*∗*^	1.5 ± 0.74	1.62 ± 0.69	433.37 ± 151.42^*∗∗*^	464.88 ± 154.9^*∗∗*^	197.04 ± 53.73	439 ± 188.76	9.02 ± 2.32	10.15 ± 2.66^*∗*^
	P-UC	10.3 ± 1.2^#^	17.25 ± 10.67^*∗∗*##^	1.62 ± 1.22	136.84 ± 38.93^##^	159.02 ± 50.35^##^	659.45 ± 440.26^#^	1139.63 ± 645.37	12.74 ± 3.45	14.3 ± 4.19

Significantly different from the normal group, ^*∗*^*P* < 0.05 and ^*∗∗*^*P* < 0.01. Significantly different from the SDDR-UC group, ^#^*P* < 0.05 and ^##^*P* < 0.01.

## Data Availability

The data used to support the findings of this study are available from the corresponding author upon request.
